# Stability of the two enveloped viruses NDV LaSota and YF-ZIKprM/E to support process development

**DOI:** 10.1371/journal.pone.0351417

**Published:** 2026-06-15

**Authors:** Sven Göbel, Lennart Jacobtorweihe, Max-Leopold Rössig, Frauke Braatz, Fabien Perugi, Yvonne Genzel, Udo Reichl

**Affiliations:** 1 Max Planck Institute for Dynamics of Complex Technical Systems, Magdeburg, Germany; 2 Technical University Brunswick, Braunschweig, Germany; 3 Valneva SE, Saint-Herblain, France; 4 Otto-von-Guericke-University, Magdeburg, Germany; Instituto Butantan, BRAZIL

## Abstract

Building on the established use of enveloped viral vectors, like lentivirus and vesicular stomatitis virus, we investigated the stability of the oncolytic Newcastle disease virus LaSota strain and the chimeric construct of a Zika vaccine candidate YF ZIKprM/E. These vectors are currently being developed for the treatment of solid tumors, such as melanoma and glioblastoma, and for vaccine initiatives, respectively. Virus stability is a critical attribute during cell culture-based virus production and also relevant for downstream processing, storage of the produced material, final vaccine storage and shelf life. Therefore, temperature and pH stability were tested as important parameters during upstream processing and freeze-thaw cycles were tested in context of laboratory-analytics. In this study, both viruses exhibited strong stability of the infectious virus titer when subjected to repeated freeze-thaw cycles. However, exposure to temperatures above 22°C substantially reduced the infectious titers, indicating sensitivity to elevated temperatures. To improve viral stability during storage, we investigated the use of sucrose as a stabilizing excipient. While this did not result in significant improvements for YF-ZIKV, an extended half-life for NDV at room temperature was observed. The observed half-life values of upstream material from NDV of 2.6 h and 2.8 h for YF-ZIKV at 37°C demand consideration of changes to the process design, such as the implementation of a perfusion process to enable continuous, cooled virus harvesting.

## Introduction

The biopharmaceutical industry is becoming increasingly dependent on viral vectors for a variety of applications, including vaccination, gene therapy, and oncolytic treatment [1,2]. Ensuring the stability of these viruses remains a central challenge throughout development, large-scale manufacturing, and long-term storage, particularly for enveloped viruses. These viruses have a lipid membrane derived from the host cell, making them vulnerable to environmental stressors [3]. It is crucial to understand the physical and chemical factors that drive viral degradation to mitigate virus instability, which can impact process yields and product quality. Throughout the manufacturing process, enveloped viruses encounter various stress conditions such as temperature, acidic or alkaline pH levels, changes in osmolality, shear force, and protease degradation. In upstream processing, standard mammalian cell culture temperatures of around 37°C [4] can significantly reduce the infectivity, as observed for measles virus (MV, half-life 1 h), severe acute respiratory syndrome coronavirus (SARS-CoV), human immunodeficiency virus (half-life 24 h) or influenza virus (half-life 13 h) [5–8]. Strategies to mitigate thermal degradation include lowering process temperatures post infection to, e.g., 33°C [9], and implementing continuous virus harvesting in perfusion mode using cell retention devices such as acoustic filters or membrane-based systems (e.g., tangential flow depth filtration) [10–12]. The latter allows for the immediate cooling of produced viruses or the addition of stabilizing agents to prevent virus degradation. Similar challenges persist during downstream processing (DSP). Multi-step operations involving filtration and chromatography expose the harvested viruses to mechanical shear, extended hold times at suboptimal temperatures, and variable solution conditions – particularly shifts in pH, osmolality and ionic strength – to which enveloped viruses are more sensitive than non-enveloped viral vectors [13–15]. For enveloped viruses such as vesicular stomatitis virus (VSV), a high pH stability under alkaline conditions but sensitivity under acidic conditions has been reported [16]. Although it is often neglected, a detailed study of stress factors such as temperature and pH value, is critical when developing a virus or viral vector production process.

This study focuses on two distinct, clinically relevant viral constructs in order to evaluate some of these parameters and their impact on the stability of enveloped viruses under processing conditions. The first is the Newcastle disease virus (NDV), which is an enveloped, negative-sense, single-stranded RNA virus with a diameter of up to 200 nm [17,18]. NDV is a well-known avian pathogen with specific lentogenic strains, particularly LaSota. These strains are gaining attention as oncolytic viruses because they can replicate selectively in tumor cells and induce an anti-tumor immune response while sparing healthy tissue [19,20]. Recent preclinical trials focused on combining NDV with other cancer therapies, as CAR-T, chemotherapy or by expressing immune checkpoint inhibitors [21–23]. In addition, NDV is under extensive preclinical and clinical investigation as vaccine vector. In the past decades, preclinical studies using NDV for the treatment of for example, AIDS, Ebola virus disease or encephalitis were conducted. Clinical trials focused on NDV as vector for SARS-CoV-2 [24].However, detailed characterization of the stability of NDV under processing conditions remains limited.

The second construct investigated in this study is a chimeric YF17D-based Zika virus vaccine candidate (YF-ZIKV) [25,26]. Flaviviruses, such as yellow fever virus (YFV) and Zika virus (ZIKV), are enveloped and contain a single-stranded RNA genome. They typically have diameters of 40–50 nm [27,28]. The YF17D vaccine is one of the most successful vaccines ever developed. Two chimeric YF17D-based vaccines are currently approved: Imojev^®^, which targets the Japanese encephalitis virus, and Dengvaxia^®^, which targets dengue viruses [29]. Further ongoing preclinical studies investigate the use of YF17D to prophylactically vaccinate against SARS-CoV2, Ebola, Lassa fever or Malaria [30]. While there is extensive literature on the stability of the formulated vaccines especially for storage and transportation, information on the stability of non-formulated infectious virus preparations during production is limited [31–34].

This study aims to provide a thorough analysis of the stability of infectious NDV and YF-ZIKV upstream material, under varying temperatures, pH levels, storage conditions, and freeze-thaw cycles. The obtained stability data could impact on sample handling and storage, but more importantly could help direct and accelerate process development towards higher yields with less losses of infectivity.

## Materials & methods

### Cell culture and virus seed

The lentogenic NDV LaSota strain was generated by reverse genetics. DNA fragments encoding the NDV genome (Genbank AF077761) and a green fluorescent protein (GFP) sequence, were inserted into a pBR322 plasmid enriched with additional restriction enzyme sites (pLaSota). The viruses were then rescued through a co-transfection of the pLaSota expression vector, and helper plasmids encoding for NDV NP, P, L and the T7 polymerase in EB66 cells [35,36]. The rescued viral particles were further produced in EB66 suspension cells (Valneva SE) using CDM4 avian medium (Cytiva, #SH31036.02) in 125 mL non-baffled shake flasks (working volume (wv)=50 mL, Corning). The EB66 cells were passaged three times per week, seeded at 3 × 10^5^ cells/mL at 37°C, 7.5% CO_2_ and 150 rpm. The cells were infected at a multiplicity of infection (MOI) of 10^-4^; 2.5 U/mL TrypLE (Thermo Fisher Scientific) was added to allow virus entry via cleavage of the fusion protein. The produced viruses were harvested 72 h post-infection (hpi) by centrifugation at 300 × g for 5 min. The supernatant was aliquoted into cryo vials and stored at −80°C. Adherent Vero E6 were used for the tissue culture infectious dose 50 (TCID_50_) assay. Vero E6 cells were cultivated in T-Flasks (Sarstedt) with GMEM medium (Thermo Fisher Scientific, #2210093) and 10% fetal calf serum (FCS, Thermo Fisher Scientific) at 5% CO_2_ and 37°C. Passages were performed twice per week.

The generation of the YF-ZIKprM/E seed virus (YF-ZIKV) has been described previously [26]. Briefly, adherent Vero E6 cells were infected at an MOI of 10^-4^ using serum-free VP-SFM medium (Thermo Fisher Scientific, #11681020) containing YF-ZIKV. The virus stock material was harvested on day 5 post-infection, then centrifuged and stored at −80°C (5.9 × 10^6^ PFU/mL). Adherent porcine stable kidney (PS) cells were used for plaque assays. PS cells were cultured in GMEM medium supplemented with LaB-M-peptone and 10% fetal calf serum (FCS) at 37°C and 5% CO_2_ in T175 cell culture flasks or 490 cm^2^ roller bottles.

### Incubation at different temperatures

The virus seed was aliquoted into cryo vials and cooled to 4°C in a fridge or heated to 33°C or 37°C using thermomixers (Eppendorf). Room temperature samples were stored separately in our laboratory with an average temperature of 22°C. At selected sampling times, three cryo vials were removed from each temperature setting and stored at −80°C for later determination of the infectious and the total virus titers.

### Incubation at different pH values

The pH levels of interest were adjusted in PBS buffer by adding NaOH or HCl (for NDV) or McIlvaine buffers and then stabilized with Tris base (for YF-ZIK). Then, 50 µL of the crude virus sample was incubated in 450 µL of the prepared pH solution for 30 min. To prevent any pH-related effects during the TCID_50_ or plaque assays, the buffer of all samples was exchanged for the respective medium using PD MiniTrap G25 columns (Cytiva). Virus recovery was assessed after column exchange and was unaffected. Single-use Mini-Traps were used according to manufacturer’s instructions. The column was equilibrated and the virus particles eluted using medium. All samples were prepared in triplicate and later measured using the corresponding virus titration assays.

### Impact of freeze-thaw cycles

A Design of Experiments (DoE) was conducted to assess the effects of freeze--thaw cycles and sucrose and sorbitol concentrations on infectious virus titer. The DoE was created in MODDE v12.1 using a central composite orthogonal (CCO) design (star distance 1.32) with three factors: number of freeze--thaw cycles (0--15), sucrose concentration (0–20%), and sorbitol concentration (0–20%). All conditions were tested in duplicate.

Freeze-thaw cycling began when aliquots were first placed in a −80°C freezer for 30 min; although the stock material had been previously frozen once, this was not counted. Tubes were thawed at room temperature on a benchtop ventilation system and refrozen immediately upon complete thawing. After completion of the assigned number of cycles, infectious titers were quantified by plaque assay. All statistical evaluations were performed using MODDE v12.1 software.

### Calculation of half-life time

To compare the different conditions, the half-life time ${\text{T}}_{\frac{\text{1}}{\text{2}}}\;$ was calculated using Equation 1. The decay constant λ was calculated based on the slope of the natural logarithm of the titer over time.


$$\begin{array}{c} {\text{T}}_{\frac{\text{1}}{\text{2}}}=\frac{\text{ln}(\text{2})}{\lambda } \end{array}$$ (1)

### Virus titration assays

The total NDV titer was determined using the HA-assay, as previously described [37]. Briefly, one sample was measured using two rows of a 96-well plate. The first row was filled with 100 µL of PBS per well and 100 µL of the sample; the second row was filled with 178.2 µL of PBS and 21.8 µL of the sample. The original sample was added to the first column and then 100 µL from each well were transferred to the next one. Subsequently, 100 µL of a chicken erythrocyte solution containing 2 × 10^7^ erythrocytes/mL (prepared using fresh blood) was added to each well. To determine the final virus titer, the wells were screened for red dots after 3 h of incubation at room temperature. The erythrocytes bind to the hemagglutinin on the viral surface. This leads to clogging of the sample, which settles to the bottom of the well and forms a red dot. The limit of detection is 2 × 10^7^ HAU/mL.

The TCID_50_ assay was used to determine the infectious NDV titers. One day prior to infection, adherent Vero E6 cells were seeded on 96 well-plates in 100 µL GMEM medium with 5% FCS and 0.5 × 10^6^ cells/mL. Additionally, 1% (v/v) gentamicin was added. On the day of infection, supernatants were discarded and washed twice with 100 µL PBS per well. Then, dilution rows were performed in GMEM with 5 U/mL trypsin (Thermo Fisher Scientific, #27250−018), without FCS. Each dilution (1.5 mL reaction tube containing 900 µL diluted sample) was transferred to one column with eight wells on a 96 well plate. After incubating for four days at 37°C in 5% CO₂, positive wells were identified with a fluorescence microscope. Due to the expression of GFP induced by the NDV LaSota strain with an eGFP insert, positive wells with NDV-infected cells emit a green light Finally, infectious virus titers were calculated using the Spearman and Kärber method [38].

Infectious YF-ZIKV titers were determined using a plaque assay with a coefficient of variance of 25% (±0.15 log) as previously described [26]. Briefly, PS cells were seeded in 24-well plates at 0.2 × 10^5^ cells per well. 48 h later, the medium was replaced with diluted virus and incubated for 4 h at 37°C. Then, a 1.6% (w/v) carboxyl-methyl-cellulose (CMC) overlay in GMEM+10% FCS was added and plates were incubated for four days at 37°C with 5% CO₂. After incubation, the supernatant was discarded, and the cells were fixed with glyoxal solution for 15 min. The cells were then stained with naphthalin black for 1 h and the plaques were manually counted. The limit of detection for both infectious virus titration methods was 3 × 10^2^ TCID_50_/mL and 588 PFU/mL, respectively.

## Results & discussion

The development of viral-based therapies, such as oncolytic viruses and live-attenuated vaccines, requires a thorough assessment of process parameters that could impact infectivity. This ensures that the therapies are both safe and effective. Stability is especially important during the development of upstream and downstream processes to prevent unnecessary virus losses. Therefore, the stability of NDV and YF-ZIKV upstream material was evaluated under various conditions, including different handling temperatures and pH-values, inactivation conditions for safety during analytics as well as freeze-thaw cycles for storage and processing steps considerations. Only the virus-containing supernatants were used, and no optimized buffer systems were employed. Likewise, the virus concentration was not varied. The obtained data were intended solely for process development, not final vaccine formulation. Clearly, our data only represent virus stability under these specific process conditions. The impact of the background buffer and medium, and cell debris containing proteolytic enzymes may result in variations in virus stability during the respective process steps. Obtained data were intended to test, whether virus stability during process development is of interest and may impact production strategies. Temperature conditions (4°C and 22°C for short-term storage; 33°C and 37°C for production) and broad pH ranges (pH 2–13) were selected to reflect relevant processing environments. Virus stability was evaluated by measuring total and/or infectious titers to determine the half-life. Moreover, tests on freeze-thaw stability were conducted, to assess the stability of virus seeds and virus sample stability for analytical assays.

### Impact of temperature on virus stability

**Fig 1:**
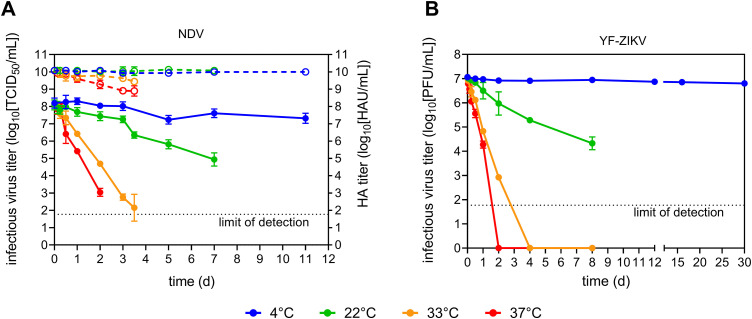
Stability of NDV and YF-ZIKV upstream material. (A) Total (empty symbols, dashed lines) and infectious NDV titer (full symbols) incubated at 4°C, 22°C, 33°C or 37°C in CDM4 medium. After incubation, infectious virus titers were determined by TCID_50_ assay. **(B)** Infectious YF-ZIKV titers after incubation in serum-free VPSFM medium. Following incubation, infectious virus titers were determined by plaque assay. All values are represented as the mean standard deviation of triplicates.

Total NDV titers remained stable during incubation at 4°C and 22°C for periods of 7 or 11 days, respectively (Fig 1). In contrast, exposure to elevated temperatures of 33°C and 37°C resulted in 4-fold and 11-fold reductions in total virus titer, respectively. The impact on infectious virus titer was more pronounced, with reductions of 7-fold at 33°C and over 1,000-fold at 37°C during the same time period. Consistent with this, the half-life of infectious virus decreased with increasing temperatures (Table 1): from 96 h at 4°C, to 17 h at 22°C, 4 h at 33°C, and 2.6 h at 37°C. Stable total virus titers suggest that NDV particles remain structurally intact even as they lose biological infectivity. Since the HA assay specifically measures the binding of surface proteins to erythrocytes, it can detect particles that are functionally attenuated but physically present. This finding is consistent with previous research indicating that the NDV LaSota strain retains structural stability at temperatures up to 72 °C [39].. This is relevant for using NDV as an inactivated vaccine in the poultry industry, where the total virus titer is used to determine the dose [40]. In contrast, a high infectious virus titer is of utmost importance for oncolytic applications. While 37°C is the standard cultivation temperature for mammalian cell culture, reducing the temperature to 33°C during the infection phase, as is often described in literature, can slow down the thermal-driven degradation and extend the half-life by 1.4 h [41,42]. Short half-life times were also observed during the production of other enveloped viruses, such as influenza or measles virus [5,41,43].

**Table 1:** Half-life of the infectious virus titer of NDV and YF-ZIKV upstream material incubated at relevant handling temperatures.

	Half-life infectious titer [h]			
	4°C	22°C	33°C	37°C
**NDV**	96.0	16.9	4.1	2.6
**YF-ZIK**	802.7	20.9	3.5	2.8

NDV YF-ZIKV were incubated at 4°C, 22°C, 33°C or 37°C. Here, the half-life corresponding to Fig 1 is illustrated.

Nevertheless, the rapid loss of infectivity at reduced production temperatures is a critical concern for batch processes exceeding 72 h, as cumulative degradation may reduce the overall infectious virus yield and alter product quality. For instance, the ratio of non-infectious to infectious particles may increase over time. Switching from batch to perfusion mode with continuous virus harvesting could reduce thermal degradation by decreasing the residence time of virus particles in the bioreactor and enabling immediate cooling and stabilization [44]. Published data on NDV stability are limited and often report short-term heat shock at elevated temperatures, rather than continuous exposure under process-relevant conditions [45,46]. Rani et al. reported that the NDV LaSota strain retained its infectivity up to 42°C, with degradation of key surface proteins only observed at 72°C. Other structural components remained stable up to 96°C after 2 h of incubation [39]. In contrast, Lomniczi et al. demonstrated a 2-log reduction in infectivity within just 2 minutes at 56°C [46].

YF-ZIKV demonstrated good stability at 4°C, with no meaningful reduction in infectious virus titer observed over a four-week period (Fig 1). However, incubation at 22°C resulted in a gradual decline in infectivity with a calculated half-life of 20.9 h. Exposure to production-relevant temperatures caused a rapid loss of infectivity with half-lives of 3.5 h at 33°C and 2.8 h at 37°C. After 24 h at 37°C or 48 h at 33°C, no infectious virus was detectable, indicating substantial degradation of viral infectivity under these production-relevant conditions. Furthermore, the use of cryoprotectants was investigated as a potential addition to the bioreactor harvest to extend the half-life of the virus. Sucrose, as well as sorbitol or trehalose, is widely recognized for its stabilizing effects on proteins and enveloped viral vectors. It primarily functions as a cryoprotectant or as a minor component in more complex formulations [47–50]. The literature especially reports on the addition of sucrose during downstream processing of virus preparations [51–53]. When we evaluated our viruses, adding 5% sucrose did not improve YF-ZIKV stability at any of the tested temperatures (S1 Fig in S1 File). However, it showed limited potential to improve short-term stability at 22°C increasing the half-life from 20.9 h to 23.0 h. Interestingly, the addition of 2.5% sucrose to NDV increased the half-life 3-fold at room temperature (S1 Table in S1 File). Further improvements may be possible if the combination of different stabilizers is studied in more detail, as has previously been shown for other viruses such as respiratory syncytial virus [54]. Compared to the parental YFV and ZIKV constructs (YFV 17D-213/77, ZIKV Rio de Janeiro state isolate [55]), the YF-ZIKV exhibited improved half-lives at 4°C and 22°C. However, its stability at production temperatures (33°C and 37°C) was up to 2-fold lower. The short half-life of YF‑ZIKV at 33–37°C underscores the necessity of continuous perfusion harvesting to allow for immediate cooling to 4°C and minimize degradation. Alternatively, harvesting prior to peak productivity could shorten the residence time and improve the overall infectious yield.

Our current study focused on the stability of the upstream material, but the stability of the virus material is equally important during downstream processing. When extrapolating our results to a larger scale and manufacturing environment, a low half-life at 22°C could present significant challenges for downstream processing. This is because viral vaccine purification often involves multiple unit operations that can expose virus particles to room temperature for several days [10]. Since the half-life of both constructs was below 1 day at 22°C, maintaining a cold-chain from virus harvesting to final formulation could be beneficial. Operating the entire downstream process at 2–8°C is difficult, especially at large scale where maintaining uniform cooling is challenging. Buffers need careful pH adjustment for cold conditions, and increased viscosity at low temperature can slow filtration and chromatography [47]. Equipment must be rated for cold-room use, and condensation control is essential. More practical strategies include cooling only the most critical unit operations, such as harvesting, concentration, or final formulation, rather than cooling the entire suite. Alternatively, moderating the cooling temperature to 18°C, for example, could significantly increase stability compared to ambient conditions while placing less demand on equipment and personnel. Finally, streamlining DSP by reducing the number of unit operations or by implementing rapid, integrated processing could drastically reduce total exposure time to adverse temperatures, thereby enhancing overall product quality and yield [56–58].

### Heat inactivation

**Fig 2:**
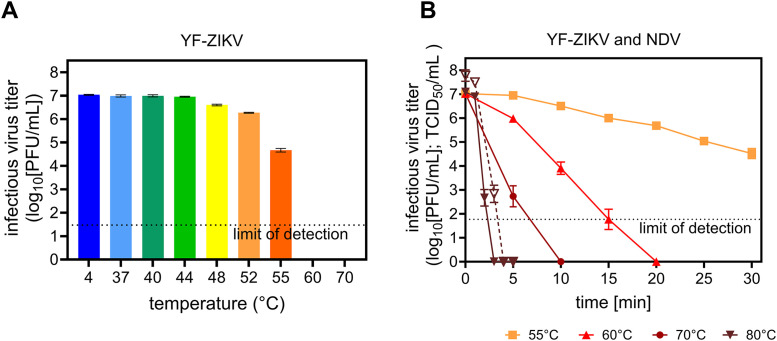
Heat inactivation of YF-ZIKV and NDV upstream material for safer laboratory handling during analytics. **(A)** The YF-ZIKV seed was incubated at various temperatures for 30 min. **(B)** Kinetics of NDV (empty symbols, dashed lines (only 80°C)) and YF-ZIKV (full symbols) inactivation at various temperatures. Following incubation, infectious virus titers were determined by TCID_50_ (NDV) and plaque assay (YF-ZIKV). All values are represented as the mean standard deviation of triplicates.

Heat inactivation is a cost-effective and practical method of reducing viral infectivity, enabling simpler and safer laboratory handling. To further assess the thermal stability of YF-ZIKV towards clearly inactivating temperatures, virus samples were incubated for 30 min at temperatures ranging from 4°C to 80°C. No infectious virus was detectable after 30 min of exposure at temperatures above 55°C (Fig 2A). To characterize the inactivation profile, the experiment was repeated with additional time-point sampling to establish an inactivation kinetics curve. Compared to other enveloped viruses, YF-ZIKV demonstrated greater thermal resistance. For example, Müller et al. (2016) reported complete inactivation of ZIKV MR766 after just 5 min at 60°C, and Zimmer et al. (2013) showed total VSV inactivation after 4 min at 55°C [16,59]. In contrast, YF-ZIKV exhibited only a minor reduction in titer (0.1 log_10_) after 5 min at 55°C, requiring incubation at 80°C to achieve similar levels of inactivation (Fig 2B). A similar heat inactivation procedure was applied to NDV. Here, infectious virus titer was still detectable after 3 min incubation at 80°C, but was completely inactivated after 5 min (Fig 2B).

### Freeze-thaw stability

**Fig 3:**
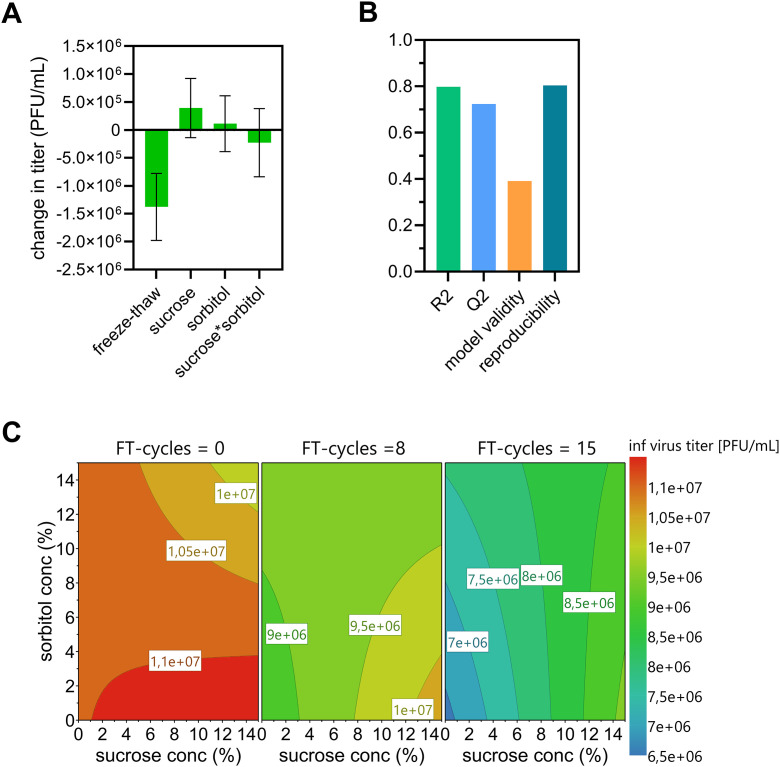
Impact of freeze-thaw cycles and addition of excipients on infectious YF-ZIKV titers. A central composite orthogonal (CCO) design of experiments (DoE) was applied. Up to 15 freeze-thaw (FT) cycles were performed, with sucrose and sorbitol concentrations tested up to 20%, and all conditions were evaluated in duplicate. **(A)** Coefficient plot. The influence of the investigated parameters on the infectious YF-ZIKV titer is shown. Positive and negative coefficients indicate the direction of the effect, while error bars represent the confidence intervals, highlighting the reliability of each parameter’s estimate. **(B)** Model statistics. **(C)** 4D response contour plot.

In addition to relevant process parameter such as temperature and pH, freeze-thaw stability was evaluated. A high tolerance to freeze-thaw cycles, is beneficial for analytic workflows, since samples can be stored or reused after sampling. As previously discussed, cryoprotectants such as sucrose and sorbitol are commonly used to stabilize infectious virus titers during storage and freeze-thaw cycles. To evaluate the impact of repeated freezing and thawing and the potential stabilizing effect of these excipients on YF-ZIKV, a central composite orthogonal design of experiments (DoE) was employed. Up to 15 freeze-thaw cycles were performed, with sucrose and sorbitol concentrations tested up to 20%. All conditions were evaluated in duplicate. This approach enabled us to verify if there are hidden synergistic effects between sucrose and sorbitol that could have been missed by traditional “one-factor-at-a-time” experiments. Multiple linear regression modeling identified the number of freeze-thaw cycles as a significant factor negatively affecting virus stability. Surprisingly, neither sucrose nor sorbitol, regardless of concentration, exhibited a statistically significant stabilizing effect on infectious virus titers (Fig 3A). The model demonstrated good performance, with an R² of 0.79, a Q² of 0.72, a model validity of 0.39, and a reproducibility of 0.80 for infectious virus titers (Fig 3B). Response contour plots were generated to visualize the model’s outcomes, showing infectious virus titers across the tested conditions (Fig 3C). Surprisingly, the titer declined by only 0.33 log_10_ after 15 freeze-thaw cycles, regardless of excipient concentration. Such extensive freeze-thawing is not expected under typical laboratory or manufacturing conditions. For comparison, YF 17D-204 exhibited a 0.4 log_10_ reduction in titer after a single freeze-thaw cycle [60]. Another study found that enveloped cytomegalovirus lost approximately 1.4 log_10_ in virus titer after just three freeze-thaw cycles in DMEM without stabilizing excipients. However, the addition of sucrose or sorbitol reduced the loss to around 0.2 log_10_ [50]. Therefore, compared to other viruses, YFV-ZIKV possesses an inherent, unexpected level of robustness and stability during freeze-thaw steps.

**Fig 4:**
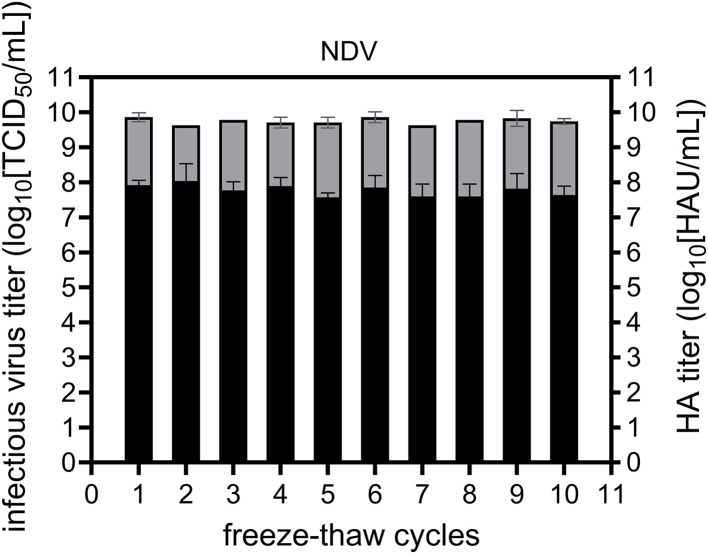
Impact of freeze-thaw cycles on NDV infectious and total titers. NDV was incubated for 15 min at −80°C and thawed at room temperature for 30 min, per cycle. Following incubation, infectious virus titers were determined by TCID_50_ (black), while HA-assay was used for total NDV titer (grey). Values are represented as the mean standard deviation of triplicates.

Moreover, NDV demonstrated equally a high tolerance to freeze-thaw cycles (Fig 4), even without the addition of further cryoprotectants. The total and infectious virus concentrations remained unchanged over the tested 10 cycles. Similarly, Levinson [61] observed no loss in infectivity for the NDV L-Kan strain after a single freeze-thaw cycle. While these findings provide a baseline for laboratory-scale handling, further studies are required to assess the impact of freezing kinetics at production volumes (e.g., 1 L or larger).

### Impact of pH value on virus stability

Although the pH value is usually tightly controlled during upstream processing, significant fluctuations can occur during DSP due to buffer exchanges, elution conditions, and concentration steps. Unit operations such as chromatography often require different pH environments, which may affect viral stability. For example, ion exchange chromatography typically operates within a pH range of 7.0–8.5. However, many affinity chromatography steps involve a brief exposure to acidic conditions (pH 2.3–3.5) during elution, followed by rapid neutralization [62–64]. Therefore, when designing a downstream process, it is crucial to assess virus stability across a broad pH range to minimize loss of infectivity and ensure overall product integrity.

To this end, NDV and YF-ZIKV were incubated in solutions adjusted to pH values ranging from pH 2–12. A 30 min incubation period at 22°C was set for all pH conditions. This standardized time accounted for the variable loading times in IEC, which are influenced by resin, column size, flow, and virus concentration as well as setup-dependent neutralization times after acidic elution in affinity chromatography. Then, MiniTrap columns were used to replace the incubation solution with the respective medium and reset the pH value to 7.

**Fig 5:**
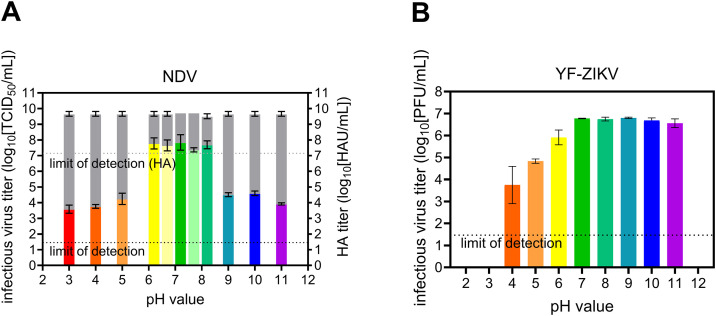
Impact of pH value on infectious titer of NDV and YF-ZIKV upstream material. NDV and YF-ZIKV were incubated for 30 min in various pH solutions. After incubation MiniTrap columns were used to exchange the media and reset the pH to 7. Following incubation, infectious virus titers were determined by TCID_50_ (NDV), or plaque assay (YF-ZIKV). Total NDV titer were measured with HA-assay. Values are represented as the mean standard deviation of triplicates.

For some NDV strains, such as Rigga or Cairo C, high pH tolerances have been reported in literature. NDV Rigga and Cairo C remained infectious after being stored up to 7 days at pH 4 [39,65]. In this study, the pH value had only a minor impact on NDV infectivity between pH 6 and 8. Although the total NDV titer did not differ between pH 3 and 11, the infectious titer was decreased 3400-fold at pH 5 and 1500-fold at pH 9, indicating a limited operational window for NDV during DSP. YF-ZIKV demonstrated stability across a broad pH range (Fig 5). Similarly to other enveloped viruses (VSV, MV, influenza A virus), stability was particularly pronounced under alkaline conditions up to pH 11 [16,66,67]. A minor reduction of 0.2 log_10_ in infectious virus titer was observed at pH 11; however, complete inactivation occurred at pH 12. Under acidic conditions, a pH-dependent decline in YF-ZIKV infectivity was observed. Lower pH values led to a greater loss of infectious virus titer. Incubation at pH 6 resulted in a 1 log₁₀ reduction in infectious virus titer, with an additional decrease of approximately 1 log₁₀ per pH unit down to pH 4. Compared to pH 7, a total reduction of 3.1 log_10_ was observed, with no infectious virus detectable below pH 4. Similar acid sensitivity has been reported for other enveloped viruses, including VSV, MV (below pH 7), YFV, and ZIKV [16,59,66,68]. For instance, YFV was completely inactivated within 1 h at pH 4.5 and ZIKV was inactivated within 10 min at pH 4 [68]. Consequently, the pH value should be tightly controlled above pH 7, to avoid virus stability-related losses. Longer pH incubation times should be investigated, as the tested periods of 30 min require a concentration step (e.g., tangential flow filtration in a real DSP) prior to chromatography loading. Depending on the capture method, incubation times can extend up to 30 h when working directly with raw virus harvests.

## Conclusions

The stability of the enveloped viruses NDV and YF-ZIKV was evaluated in the context of an integration of production and downstream processing. Unlike NDV, YF-ZIKV showed high tolerance to alkaline conditions. However, both viruses showed a reduction in infectious virus titer at pH values below 6. During upstream production, the pH value tends to shift towards acidity due to lactate and CO_2_ accumulation. Therefore, strategies such as tight pH control, base addition, and management of dissolved CO_2_ should be implemented to maintain a neutral pH and preserve virus stability.

Both viruses tolerated at least ten freeze-thaw cycles without a meaningful loss of infectivity, enabling simpler analytic workflows. However, temperature clearly impacted the infectious virus titer of both viruses. Their half-life at the typical process temperature (37°C) indicates a need for continuous cooling (4°C) of virus harvests until downstream processing. For NDV vaccine applications, where infectious titers are less critical, thermal degradation may play a smaller role because the total virus titer remained comparatively stable. Overall, the stability of the infectious virus particles directly influences the design of both upstream and downstream processes, requiring careful adaptation of the process conditions to preserve product quality and yield.

## Supporting information

S1 File Supporting Information_new. (DOCX)

## References

[1] Douglas RG, Samant VB. The vaccine industry. Plotkin’s Vaccines. 2018. 41-50.e1.

[2] Moleirinho MG, Silva RJS, Alves PM, Carrondo MJT, Peixoto C. Current challenges in biotherapeutic particles manufacturing. Expert Opin Biol Ther. 2020;20(5):451–65. doi: 10.1080/14712598.2020.1693541 31773998

[3] Firquet S, et al., Survival of enveloped and non-enveloped viruses on inanimate surfaces. Microbes Environ, 2015. 30(2): 140–4. 25843687 10.1264/jsme2.ME14145 PMC4462923

[4] Sandell L, Sakai D. Mammalian cell culture. Current Protocols Essential Laboratory Techniques. 2011.

[5] Weiss. Influence of process conditions on measles virus stability. American Journal of Biochemistry and Biotechnology. 2013;9(3):243–54. doi: 10.3844/ajbbsp.2013.243.254

[6] Chan KH, Peiris JSM, Lam SY, Poon LLM, Yuen KY, Seto WH. The effects of temperature and relative humidity on the viability of the SARS coronavirus. Adv Virol. 2011;2011:734690. doi: 10.1155/2011/734690 22312351 PMC3265313

[7] Yang G, Ojha CR, Russell CJ. Relationship between hemagglutinin stability and influenza virus persistence after exposure to low pH or supraphysiological heating. PLoS Pathog. 2021;17(9):e1009910. doi: 10.1371/journal.ppat.1009910 34478484 PMC8445419

[8] Tjøtta E, Hungnes O, Grinde B. Survival of HIV-1 activity after disinfection, temperature and pH changes, or drying. J Med Virol. 1991;35(4):223–7. doi: 10.1002/jmv.1890350402 1802953

[9] Wechuck JB, Ozuer A, Goins WF, Wolfe D, Oligino T, Glorioso JC, et al. Effect of temperature, medium composition, and cell passage on production of herpes-based viral vectors. Biotechnol Bioeng. 2002;79(1):112–9. doi: 10.1002/bit.10310 17590937

[10] Göbel S, Pelz L, Silva CAT, Brühlmann B, Hill C, Altomonte J, et al. Production of recombinant vesicular stomatitis virus-based vectors by tangential flow depth filtration. Appl Microbiol Biotechnol. 2024;108(1):240. doi: 10.1007/s00253-024-13078-6 38413399 PMC10899354

[11] Castilho LR, Medronho RA. Cell retention devices for suspended-cell perfusion cultures. Adv Biochem Eng Biotechnol. 2002;74:129–69. doi: 10.1007/3-540-45736-4_7 11991177

[12] Jacobtorweihe L, et al. High cell density perfusion process of quail cells producing oncolytic rVSV-NDV. Eng Life Sci. 2025;25(7):e70035. 10.1002/elsc.70035 PMC12256980 40661158

[13] Singh N, Heldt CL. Challenges in downstream purification of gene therapy viral vectors. Current Opinion in Chemical Engineering. 2022;35:100780. doi: 10.1016/j.coche.2021.100780

[14] Fernandes RP, Nestola P, Peixoto C. Downstream processing of viral-based vaccines. Bioprocessing of Viral Vaccines. CRC Press. 2022. p. 175–99. doi: 10.1201/9781003229797-7

[15] Fernandes RP, Göbel S, Reiter M, Bryan A, Altomonte J, Genzel Y, et al. Streamlining the purification of a clinical-grade oncolytic virus for therapeutic applications. Separation and Purification Technology. 2025;354:128769. doi: 10.1016/j.seppur.2024.128769

[16] Zimmer B, Summermatter K, Zimmer G. Stability and inactivation of vesicular stomatitis virus, a prototype rhabdovirus. Vet Microbiol. 2013;162(1):78–84. doi: 10.1016/j.vetmic.2012.08.023 22995872

[17] Sinkovics JG, Horvath JC. Newcastle disease virus (NDV): Brief history of its oncolytic strains. J Clin Virol. 2000;16(1):1–15. doi: 10.1016/s1386-6532(99)00072-4 10680736

[18] Burman B, Pesci G, Zamarin D. Newcastle disease virus at the forefront of cancer immunotherapy. Cancers (Basel). 2020;12(12):3552. doi: 10.3390/cancers12123552 33260685 PMC7761210

[19] Zamarin D, Palese P. Oncolytic Newcastle disease virus for cancer therapy: Old challenges and new directions. Future Microbiol. 2012;7(3):347–67. doi: 10.2217/fmb.12.4 22393889 PMC4241685

[20] Sun J, Wang J, Xiao M, Chen L, Guan Y. Research progress on recombinant NDV in cancer therapy. Front Immunol. 2025;16:1735440. doi: 10.3389/fimmu.2025.1735440 41479917 PMC12753920

[21] Wang M, Jiang K, Aicher A, Heeschen C. Engineering the tumor microenvironment: Oncolytic NDV to facilitate CAR-T cell therapy. J Transl Med. 2025;23(1):1316. doi: 10.1186/s12967-025-07342-0 41257772 PMC12628613

[22] Sabri H, Barough MS, Zafari E, Pakjoo M, Mahdavi M, Esmaeili F, et al. Synergistic anti-tumor effects of newcastle disease virus and doxorubicin: Evidence from a murine breast cancer model. Int Immunopharmacol. 2024;143(Pt 2):113481. doi: 10.1016/j.intimp.2024.113481 39467343

[23] Vijayakumar G, McCroskery S, Palese P. Engineering newcastle disease virus as an oncolytic vector for intratumoral delivery of immune checkpoint inhibitors and immunocytokines. J Virol. 2020;94(3):e01677-19. doi: 10.1128/JVI.01677-19 31694938 PMC7000961

[24] Fulber JPC, Kamen AA. Development and scalable production of newcastle disease virus-vectored vaccines for human and veterinary use. Viruses. 2022;14(5):975. doi: 10.3390/v14050975 35632717 PMC9143368

[25] Kum DB, Boudewijns R, Ma J, Mishra N, Schols D, Neyts J, et al. A chimeric yellow fever-Zika virus vaccine candidate fully protects against yellow fever virus infection in mice. Emerg Microbes Infect. 2020;9(1):520–33. doi: 10.1080/22221751.2020.1730709 32116148 PMC7067203

[26] Göbel S, Kazemi O, Ma J, Jordan I, Sandig V, Paulissen J, et al. Parallel multifactorial process optimization and intensification for high-yield production of live YF17D-vectored zika vaccine. Vaccines (Basel). 2024;12(7):755. doi: 10.3390/vaccines12070755 39066393 PMC11281342

[27] Heinz FX, Stiasny K. Flaviviruses and flavivirus vaccines. Vaccine. 2012;30(29):4301–6. doi: 10.1016/j.vaccine.2011.09.114 22682286

[28] Schmaljohn AL, D M, Baron S. Alphaviruses (Togaviridae) and Flaviviruses (Flaviviridae). Medical Microbiology. 4th ed. Galveston (TX): University of Texas Medical Branch at Galveston. 1996. 21413253

[29] Theiler M, Smith HH. The use of yellow fever virus modified by in vitro cultivation for human immunization. J Exp Med. 1937;65(6):787–800. doi: 10.1084/jem.65.6.787 19870634 PMC2133527

[30] Sanchez-Felipe L, Alpizar YA, Ma J, Coelmont L, Dallmeier K. YF17D-based vaccines - standing on the shoulders of a giant. Eur J Immunol. 2024;54(5):e2250133. doi: 10.1002/eji.202250133 38571392

[31] Ishak R, Howard CR. The thermal stability of yellow fever vaccines. Mem Inst Oswaldo Cruz. 1990;85(3):339–45. doi: 10.1590/s0074-02761990000300011 2134708

[32] Meister TL, Frericks N, Kleinert RDV, Rodríguez E, Steinmann J, Todt D, et al. Inactivation of yellow fever virus by WHO-recommended hand rub formulations and surface disinfectants. PLoS Negl Trop Dis. 2024;18(6):e0012264. doi: 10.1371/journal.pntd.0012264 38900788 PMC11218936

[33] Barban V, Girerd Y, Aguirre M, Gulia S, Pétiard F, Riou P, et al. High stability of yellow fever 17D-204 vaccine: a 12-year restrospective analysis of large-scale production. Vaccine. 2007;25(15):2941–50. doi: 10.1016/j.vaccine.2006.06.082 16914238

[34] Monath TP. Stability of yellow fever vaccine. Dev Biol Stand. 1996;87:219–25. 8854020

[35] Peeters BP, de Leeuw OS, Koch G, Gielkens AL. Rescue of Newcastle disease virus from cloned cDNA: Evidence that cleavability of the fusion protein is a major determinant for virulence. J Virol. 1999;73(6):5001–9. doi: 10.1128/JVI.73.6.5001-5009.1999 10233962 PMC112544

[36] Römer-Oberdörfer A, Mundt E, Mebatsion T, Buchholz UJ, Mettenleiter TC. Generation of recombinant lentogenic Newcastle disease virus from cDNA. J Gen Virol. 1999;80 (Pt 11):2987–95. doi: 10.1099/0022-1317-80-11-2987 10580061

[37] Kalbfuss B, Knöchlein A, Kröber T, Reichl U. Monitoring influenza virus content in vaccine production: precise assays for the quantitation of hemagglutination and neuraminidase activity. Biologicals. 2008;36(3):145–61. doi: 10.1016/j.biologicals.2007.10.002 18561375

[38] Hierholzer JC, Killington RA. Virus isolation and quantitation. Virology Methods Manual. Elsevier. 1996. 25–46. doi: 10.1016/b978-012465330-6/50003-8

[39] Rani S, Gogoi P, Kumar S. Spectrum of Newcastle disease virus stability in gradients of temperature and pH. Biologicals. 2014;42(6):351–4. doi: 10.1016/j.biologicals.2014.08.006 25284348

[40] Wang G, Zhu R, Yang L, Wang K, Zhang Q, Su X, et al. Non-thermal plasma for inactivated-vaccine preparation. Vaccine. 2016;34(8):1126–32. doi: 10.1016/j.vaccine.2015.10.099 26529075

[41] Zinnecker T, Thiele K, Schmidberger T, Genzel Y, Reichl U. Influenza a virus production following quality by design principles. Eng Life Sci. 2025;25(4):e70027. doi: 10.1002/elsc.70027 40271119 PMC12016631

[42] Fulber JPC, Farnós O, Kiesslich S, Yang Z, Dash S, Susta L, et al. Process development for newcastle disease virus-vectored vaccines in serum-free vero cell suspension cultures. Vaccines (Basel). 2021;9(11):1335. doi: 10.3390/vaccines9111335 34835266 PMC8623276

[43] Weiss K, Gerstenberger J, Salzig D, Mühlebach MD, Cichutek K, Pörtner R, et al. Oncolytic measles viruses produced at different scales under serum‐free conditions. Engineering in Life Sciences. 2015;15(4):425–36. doi: 10.1002/elsc.201400165

[44] Tapia F, Vázquez-Ramírez D, Genzel Y, Reichl U. Bioreactors for high cell density and continuous multi-stage cultivations: options for process intensification in cell culture-based viral vaccine production. Appl Microbiol Biotechnol. 2016;100(5):2121–32. doi: 10.1007/s00253-015-7267-9 26758296 PMC4756030

[45] Wen G, Hu X, Zhao K, Wang H, Zhang Z, Zhang T, et al. Molecular basis for the thermostability of Newcastle disease virus. Sci Rep. 2016;6:22492. doi: 10.1038/srep22492 26935738 PMC4776148

[46] Lomniczi B. Thermostability of Newcastle disease virus strains of different virulence. Arch Virol. 1975;47(3):249–55. doi: 10.1007/BF01317812 1168045

[47] Croyle MA, Cheng X, Wilson JM. Development of formulations that enhance physical stability of viral vectors for gene therapy. Gene Ther. 2001;8(17):1281–90. doi: 10.1038/sj.gt.3301527 11571564

[48] Cruz PE, Silva AC, Roldão A, Carmo M, Carrondo MJT, Alves PM. Screening of novel excipients for improving the stability of retroviral and adenoviral vectors. Biotechnol Prog. 2006;22(2):568–76. doi: 10.1021/bp050294y 16599578

[49] Evans RK, Nawrocki DK, Isopi LA, Williams DM, Casimiro DR, Chin S, et al. Development of stable liquid formulations for adenovirus-based vaccines. J Pharm Sci. 2004;93(10):2458–75. doi: 10.1002/jps.20157 15349956

[50] Kumru OS, Saleh-Birdjandi S, Antunez LR, Sayeed E, Robinson D, van den Worm S, et al. Stabilization and formulation of a recombinant Human Cytomegalovirus vector for use as a candidate HIV-1 vaccine. Vaccine. 2019;37(44):6696–706. doi: 10.1016/j.vaccine.2019.09.027 31548012 PMC6863464

[51] Nguyen HM, et al. Growth, purification, and titration of oncolytic herpes simplex virus. J Vis Exp. 2021;171. 10.3791/62677 PMC8447238 34057449

[52] Leigheb S, Rahimzadeh M, Virgolini N, Tleuova A, Mousavian M, Dürauer A, et al. Capture of adeno‐associated viruses from clarified lysate by continuous flow ultracentrifugation: A comparative study of iodixanol and sucrose gradients. J of Chemical Tech & Biotech. 2025. doi: 10.1002/jctb.7901

[53] Bakhshizadeh Gashti A, Chahal PS, Gaillet B, Garnier A. Purification of recombinant vesicular stomatitis virus-based HIV vaccine candidate. Vaccine. 2023;41(13):2198–207. doi: 10.1016/j.vaccine.2023.02.058 36842887

[54] Gupta CK, Leszczynski J, Gupta RK, Siber GR. Stabilization of respiratory syncytial virus (RSV) against thermal inactivation and freeze-thaw cycles for development and control of RSV vaccines and immune globulin. Vaccine. 1996;14(15):1417–20. doi: 10.1016/s0264-410x(96)00096-5 8994316

[55] Nikolay A. Intensified yellow fever and Zika virus production in animal cell culture. Otto-von-Guericke University Magdeburg. 2020.

[56] Gomis-Fons J, Löfgren A, Andersson N, Nilsson B, Berghard L, Wood S. Integration of a complete downstream process for the automated lab-scale production of a recombinant protein. J Biotechnol. 2019;301:45–51. doi: 10.1016/j.jbiotec.2019.05.013 31145936

[57] Müller JM, Müllerová T, Tobler D, Hauri D, Plieninger R, Higuchi Y, et al. Enrichment of Full AAV2 Using Multicolumn Countercurrent Solvent Gradient Purification (MCSGP). Biotechnol Bioeng. 2025;122(9):2420–32. doi: 10.1002/bit.29036 40468522

[58] Gerstweiler L, Billakanti J, Bi J, Middelberg APJ. An integrated and continuous downstream process for microbial virus-like particle vaccine biomanufacture. Biotechnol Bioeng. 2022;119(8):2122–33. doi: 10.1002/bit.28118 35478403 PMC9542101

[59] Muller JA, et al. Inactivation and environmental stability of Zika virus. Emerg Infect Dis. 2016;22(9):1685–7. 27367466 10.3201/eid2209.160664 PMC4994368

[60] Sood DK, Aggarwal RK, Sharma SB, Sokhey J, Singh H. Study on the stability of 17D-204 yellow fever vaccine before and after stabilization. Vaccine. 1993;11(11):1124–8. doi: 10.1016/0264-410x(93)90073-7 8249431

[61] Levinson W. Inactivation of Newcastle disease virus by freezing in the presence of guanidine hydrochloride. Virology. 1965;27(4):559–65. doi: 10.1016/0042-6822(65)90181-9 5855571

[62] Njayou M, Quash G. Purification of measles virus by affinity chromatography and by ultracentrifugation: A comparative study. J Virol Methods. 1991;32(1):67–77. doi: 10.1016/0166-0934(91)90186-4 1648573

[63] Chu W, Shastry S, Barbieri E, Prodromou R, Greback-Clarke P, Smith W, et al. Peptide ligands for the affinity purification of adeno-associated viruses from HEK 293 cell lysates. Biotechnol Bioeng. 2023;120(8):2283–300. doi: 10.1002/bit.28495 37435968 PMC10440015

[64] Mendes JP, Bergman M, Solbrand A, Peixoto C, Carrondo MJT, Silva RJS. Continuous affinity purification of adeno-associated virus using periodic counter-current chromatography. Pharmaceutics. 2022;14(7):1346. doi: 10.3390/pharmaceutics14071346 35890242 PMC9323845

[65] Tolba MK, Eskarous JK. pH-Stability patterns of some strains of Newcastle disease and fowl-plague viruses. Arch Mikrobiol. 1959;34:333–8. doi: 10.1007/BF00447095 13838681

[66] Eckhardt D, Dieken H, Loewe D, Grein TA, Salzig D, Czermak P. Purification of oncolytic measles virus by cation-exchange chromatography using resin-based stationary phases. Separation Science and Technology. 2021;57(6):886–96. doi: 10.1080/01496395.2021.1955267

[67] Seguchi M, et al. Effects of alkaline solutions on the structure and function of influenza A virus. Viruses. 2024;16(10). 10.3390/v16101636 PMC11512367 39459968

[68] Pato TP, Souza MCO, Silva ANMR, Pereira RC, Silva MV, Caride E, et al. Development of a membrane adsorber based capture step for the purification of yellow fever virus. Vaccine. 2014;32(24):2789–93. doi: 10.1016/j.vaccine.2014.02.036 24631080

