# Supplementary material for: Stability of the two enveloped viruses NDV LaSota and YF-ZIKprM/E to support process development
Source: PLoS One. 2026 Jun 15;21(6):e0351417. doi: 10.1371/journal.pone.0351417 (PMC13268197; doi:10.1371/journal.pone.0351417)
Supplement: S1 File — (DOCX) [file pone.0351417.s001.docx]

**Supporting information**

**S1 Table:** **Half-life of the infectious NDV titer incubated at 22°C, with and without the addition of sucrose.**

| **Sucrose concentration [%]** | 0 | 2.5 | 5 | 10 |
| --- | --- | --- | --- | --- |
| **Half-life [h]** | 16.9 | 56.4 | 36.7 | 59.9 |


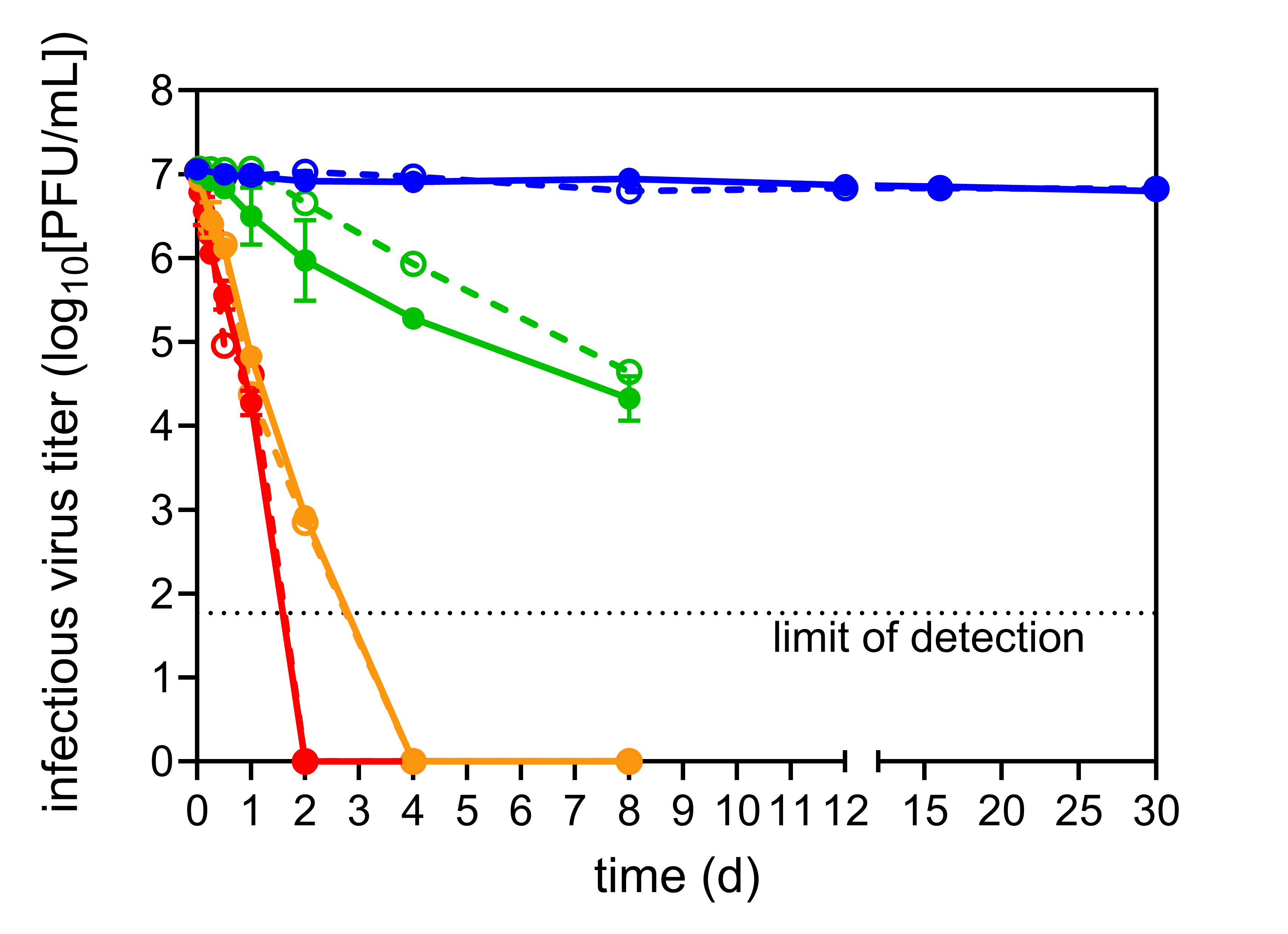


**S1 Fig:** **Infectious YF-ZIKV titers after incubation in serum-free VPSFM medium containing either no sucrose (full symbols) or 5 % (v/v) sucrose (empty symbols, dashed lines).**

Following incubation at 4°C (●), 22°C (●), 33°C (●) and 37°C (●), infectious virus titers were determined by plaque assay. All values are represented as the mean ± standard deviation of triplicates.

**S2 Table:** **Summary of the Design of Experiments (DoE) approach for YF-ZIK using multiple linear regression.**

|  | **Coeff. SC** | **Std. Err.** | **P** | **Conf. int(±)** |
| --- | --- | --- | --- | --- |
| Constant | 9.31E6 | 165551 | 0 | 332865 |
| Suc | 390000 | 530000 | 0.0813818 | 403127 |
| Sor | 110000 | 500000 | 0.454312 | 409317 |
| FT | -1380000 | 600000 | 0.004724 | 422904 |
| Suc*Sor | -230000 | 610000 | 0.0512 | 457044 |
| N = 53 | Q^2^ = 0.724 |  | Cond. no. = | 1.397 |
| DF = 48 | R^2^ = 0.798 |  | RSD = | 1.2E6 |
|  | MV = 0.393 |  | Coinfidence = | 0.95 |

Coeff. SC: Scaled and Centered Coefficients.

Std. Err.: Standard Error of the coefficient.

P: p-value.

Conf. int(±): 95% Confidence Interval for the coefficients.

Suc: Sucrose concentration.

Sor: Sorbitol concentration.

FT: Number of Freeze-Thaw cycles.

Suc*Sor: Interaction effect between Sucrose and Sorbitol.

N: Total number of observations (53).

DF: Degrees of Freedom (48).

R2 / R2 adj.: Coefficient of determination and adjusted R2.

Q2: Estimate of the model's predictive ability.

RSD: Residual Standard Deviation.

Cond. no.: Condition number.

MV: model validity
